# Potentially suitable habitats of *Daodi* goji berry in China under climate change

**DOI:** 10.3389/fpls.2023.1279019

**Published:** 2024-01-09

**Authors:** Jianling Li, Changrong Deng, Guozhen Duan, Zhanlin Wang, Yede Zhang, Guanghui Fan

**Affiliations:** ^1^ Academy of Agriculture and Forestry Sciences, Qinghai University, Xining, China; ^2^ Qinghai Plateau Tree Genetics and Breeding Laboratory, Qinghai University, Xining, China; ^3^ State Key Laboratory of Plateau Ecology and Agriculture, Qinghai University, Xining, China; ^4^ Laboratory for Research and Utilization of Qinghai Tibet Plateau Germplasm Resources, Qinghai University, Xining, China; ^5^ Qinghai Kunlun Goji Industry Technology Innovation Research Co., Ltd., Delingha, China

**Keywords:** *Lycium barbarum*, *Daodi*, MaxEnt, potential distribution, climate change

## Abstract

**Introduction:**

Goji berry (*Lycium barbarum* L.) is a famous edible and medicinal herb worldwide with considerable consumption. The recent cultivation of goji berries in the *Daodi* region was seriously reduced due to increased production costs and the influence of policy on preventing nongrain use of arable land in China. Consequently, production of *Daodi* goji berry was insufficient to meet market demands for high-quality medicinal materials. Searching for regions similar to the *Daodi* region was necessary.

**Methods:**

The MaxEnt model was used to predicted the current and future potential regions suitable for goji berry in China based on the environmental characteristics of the *Daodi* region (including Zhongning County of Zhongwei prefecture-level city, and its surroundings), and the ArcGIS software was used to analyze the changes in its suitable region.

**Results:**

The results showed that when the parameters were FC = LQHP and RM = 2.1, the MaxEnt model was optimal, and the AUC and TSS values were greater than 0.90. The mean temperature and precipitation of the coldest quarter were the most critical variables shaping the distribution of *Daodi* goji berries. Under current climate conditions, the suitable habitats of the *Daodi* goji berry were 45,973.88 km^2^, accounting for 0.48% of China’s land area, which were concentrated in the central and western Ningxia Province (22,589.42 km^2^), and the central region of Gansu Province (18,787.07 km^2^) bordering western Ningxia. Under future climate scenarios, the suitable area was higher than that under current climate conditions and reached the maximum under RCP 6.0 (91,256.42 km^2^) in the 2050s and RCP 8.5 (82,459.17 km^2^) in the 2070s. The expansion regions were mainly distributed in the northeast of the current suitable ranges, and the distributional centroids were mainly shifted to the northeast. The moderately and highly suitable overlapping habitats were mainly distributed in Baiyin (7,241.75 km^2^), Zhongwei (6,757.81 km^2^), and Wuzhong (5, 236.87 km^2^) prefecture-level cities.

**Discussion:**

In this stduy, MaxEnt and ArcGIS were applied to predict and analyze the suitable habitats of Daodi goji berry in China under climate change. Our results indicate that climate warming is conducive to cultivating *Daodi* goji berry and will not cause a shift in the *Daodi* region. The goji berry produced in Baiyin could be used to satisfy the demand for high-quality medicinal materials. This study addresses the insufficient supply and guides the cultivation of Daodi goji berry.

## 1 Introduction

Chinese medicinal materials (CMMs) are the foundation of traditional Chinese medicine (TCM) for disease prevention and treatment, and their quality is closely related to the safety and efficacy of TCM (Wang et al., 2015; Lu et al., 2022). Therefore, more than 60% of CMMs included in the Chinese Pharmacopoeia (2020 edition) must be capable of detecting chemical component concentrations (Xu et al., 2021). However, CMMs have multitudinous pharmacology components that play considerable roles in physiological regulation through synergistic, antagonistic, or unilateral effects. CMMs cannot rely on several mandatory detected components to assess their quality due to the limitations of science and technology (Gao et al., 2011; Wu et al., 2018). Evaluating the comprehensive quality of CMMs based on clinical efficacy has long been a major scientific issue for TCM (Fang et al., 2022). In ancient China, based on the abundant clinical efficacy of CMMs from different regions for long-term practice, TCM practitioners gradually proposed the concept of *Daodi* CMMs (also known as *Didao*, authentic, genuine, geoauthentic medicinal materials, etc.) (Zhao et al., 2012; Yang et al., 2018). According to the Law of the People’s Republic of China on TCM implemented in 2017, *Daodi* CMMs refer to those selected through long-term clinical applications of TCM and produced in specific geographic regions, with better quality and efficacy than others. Thus, *Daodi* CMMs are widely recognized and have enjoyed a good reputation in the TCM industry for centuries.

TCM plays an ever-increasing role in modern medicine and healthcare, gradually gaining recognition worldwide (Gao et al., 2011). The cultivation area of CMMs has increased enormously with the increasing demand for TCM services. In 2020, the total planting area of CMMs exceeded 56,000 km^2^ in China (Wang et al., 2022a). However, the synthesis of plant bioactive constituents (mainly secondary metabolites) is influenced by external environmental conditions, such as climate and soil (Ncube et al., 2012; Lei et al., 2018). Different regional environments lead to an uneven quality of CMMs (Liu et al., 2020). In recent years, the Chinese government has issued several documents advocating for enterprises to use *Daodi* CMMs to ensure the quality of CMMs and the efficacy of TCMs (Standing Committee of the National People's Congress of China, 2019; National Medical Products Administration of China, 2020; National Medical Products Administration of China et al., 2022). However, the supply of CMMs in the *Daodi* regions is limited, making it challenging to meet the considerable demands of the market. Meanwhile, the Chinese government released a guideline on preventing nongrain use of arable land and stabilizing grain production in 2020 (General Office of the State Council of China, 2020). Such a policy severely restricted the cultivation of CMMs. In 2022, the cultivation area of CMMs decreased by 6.82% compared with 2021 (Insight and Info, 2022), leading to a decrease in the supply of *Daodi* CMMs.

Even worse, the surface temperature on Earth has increased by 0.85°C over the past 130 years and is predicted to rise continuously due to increased greenhouse gas emissions (Stocker, 2013). The climate has critically impacts on plant growth, development, and distribution. Many plants are expected to shift their geographical ranges for survival in response to global warming (Thuiller, 2007). Therefore, climate change may lead to the shift of CMMs’ suitable ranges. For example, the suitable area of wild jujube [Z*iziphus jujuba* Mill. var. *spinosa* (Bunge) Hu ex H. F. Chou] in the future was found to increase except for SSP1-2.6 and SSP5-8.5 in the 2070s, and SSP5-8.5 in the 2090s, and the centroids of its suitable region shifted to the northwest (Zhao et al., 2022a). The suitable habitats of shiny-leaf prickly-ash [*Zanthoxylum nitidum* (Roxb.) DC.] in the 2050s and 2070s were predicted to gradually reduce southward under different greenhouse gas emission modes (Yang et al., 2023). The formation of high-quality *Daodi* CMMs is closely related to their growing environments (Zhao et al., 2012; Yang et al., 2018; Liu et al., 2020). Therefore, drastic climate change is likely to result in a shift in the location of *Daodi* regions. It has been proved that the *Daodi* region of oriental water plantain [*Alisma orientalis* (Sam.) Juzep.] has shifted from Hanzhong, Shaanxi Province, in the Song Dynasty to Jianning, Fujian Province, in the Qing Dynasty due to the cooling climate (Peng et al., 2013). Under climate warming, the original *Daodi* region may no longer be suitable for the distribution of CMMs. However, few studies have focused on the influence of global warming on changes in *Daodi* regions. Understanding the impact of climate change on the distribution of *Daodi* CMMs is of great significance to their cultivation and conservation.

**Figure 1:**
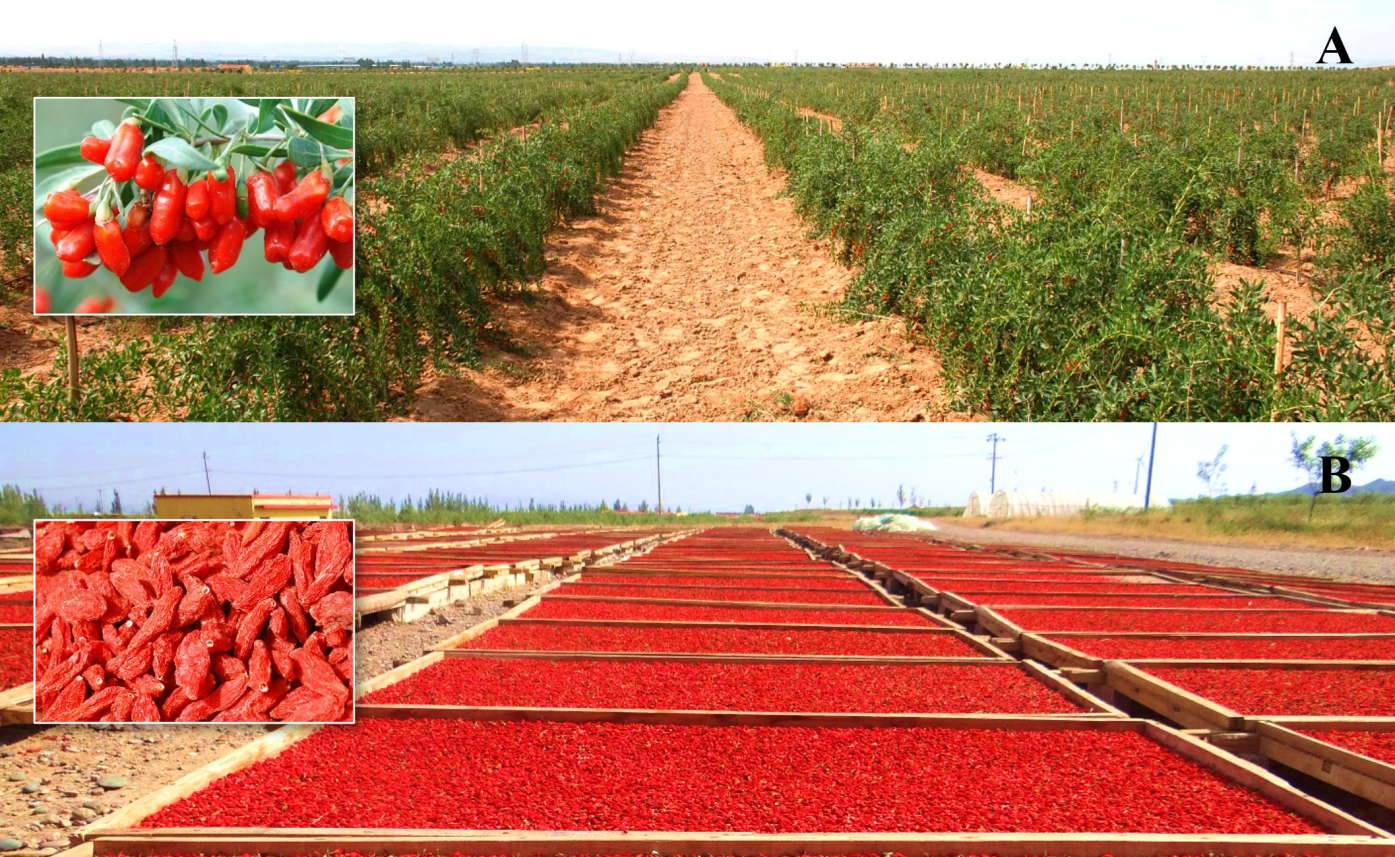
**(A)** The production base of goji berry in the *Daodi* region and **(B)** drying processing of the *Daodi* goji berry.

The goji berry (*Lycium barbarum* L.) is a significant medicinal and edible herb with the efficacy of nourishing the liver and kidney, benefiting essence, and improving eyesight (**Figure 1**) (Ma et al., 2022; Vidović et al., 2022). The annual demand for the herb exceeded 300,000 tons, and the cultivation area covered over 1,200 km^2^ (Wang et al., 2019a; Ma et al., 2021). These numbers made this species one of the most consumed and cultivated CMMs in China. In recent years, the cultivation area of goji berry in the *Daodi* region (Zhongning County and its surrounding areas) has decreased to less than 10% of that of China due to increased production costs and the policy of preventing the nongrain use of arable land. Thus, the production in the *Daodi* region is insufficient to meet market demands. Goji berries from other regions are transported to Zhongning County to disguise *Daodi* CMMs for sale to meet the considerable market demands (Lv, 2019). As the designated ecological environments are the limited factors influencing the formation of *Daodi* CMMs, leading to the quality difference between *Daodi* and non-*Daodi* CMMs (Zhao et al., 2012; Liu et al., 2020), screening similar production areas based on the environmental characteristics of the *Daodi* regions is of great significance in meeting the demands of food, Chinese patent medicines, formula granules, decoction pieces, etc. for high-quality goji berry.

Species distribution models (SDMs) are powerful tools for predicting the potential geographical distribution of species based on currently known species distribution records and the corresponding site characteristics, which have been widely used in biological introduction and cultivation, species protection, and invasive species prevention (Santana et al., 2019; Zhao et al., 2022a; Yang et al., 2023). Various SDMs, such as Bioclim, Climex, Domain, GARP, and MaxEnt, have been developed to predict the potential distribution of species (Srivastava et al., 2020; Li et al., 2023). The MaxEnt model stands out for its high prediction accuracy with incomplete or small sample datasets, short running time, and easy operation (Pearson et al., 2007; Ortega-Huerta and Townsend Peterson, 2008).

Although the impact of climate change on the distribution of goji berries in China has been previously predicted using the MaxEnt model (Wang et al., 2022b), its effects on *Daodi* goji berries were still unclear. In this study, the potentially suitable habitats of goji berries under current and future climate scenarios were predicted based on the environmental characteristics of the *Daodi* region using the optimized MaxEnt model. The major environmental variables influencing its distribution were identified, and the changes in its distribution were analyzed. Our study will hopefully provide guidance for scientific cultivation and contribute to solving the supply shortage of high-quality goji berries.

## 2 Materials and methods

### 2.1 Materials

#### 2.1.1 Distribution points of *Daodi* goji berry and environmental variables

**Figure 2:**
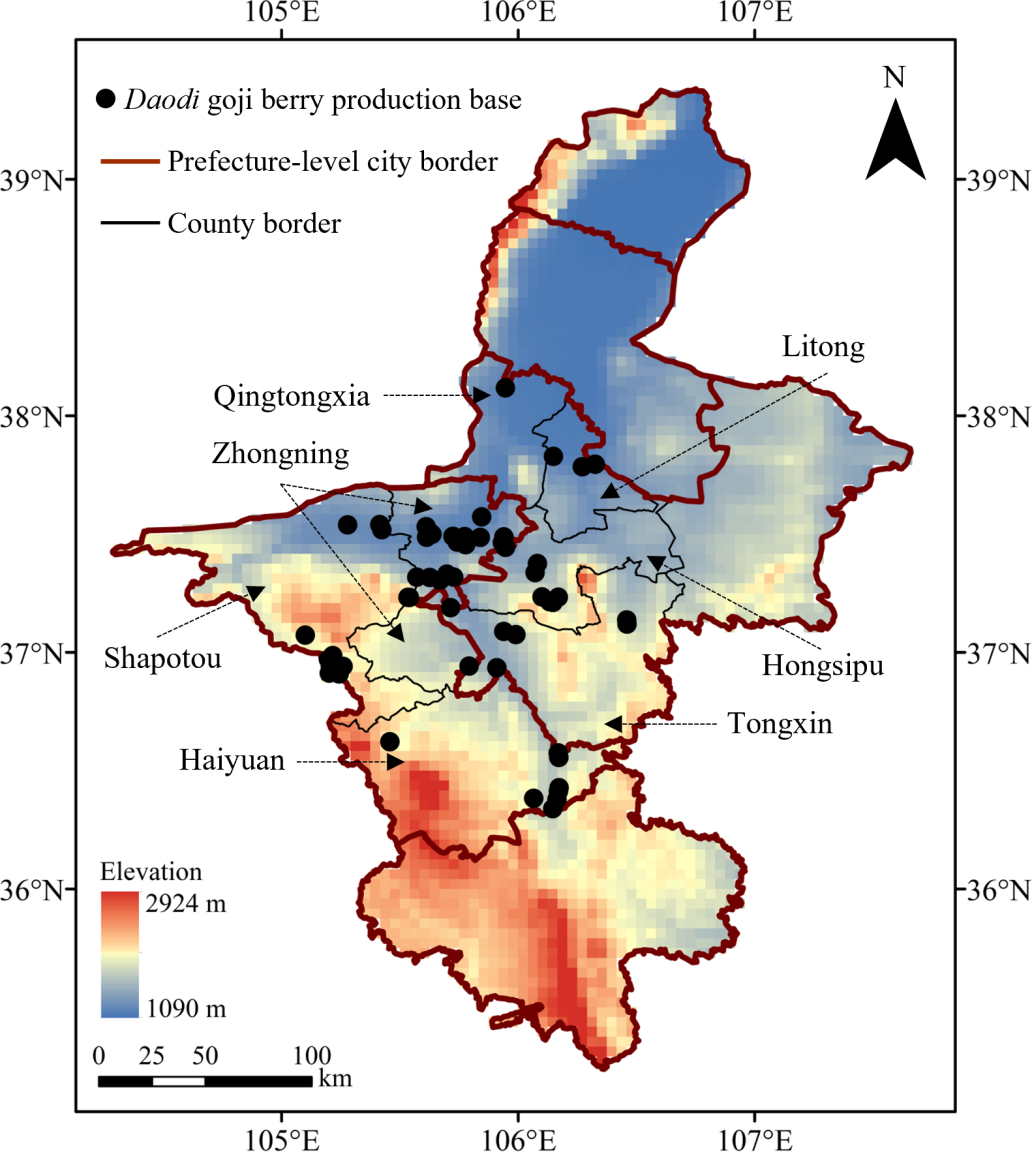
Distribution information of goji berry planting bases in the *Daodi* region of Ningxia Province.

According to the standard for *Daodi* medicinal materials, issued by the Chinese Society of TCM (CACM) in 2019, the *Daodi* region of goji berry is located in Zhongning County and its surrounding areas (Huang et al., 2020). From 2019 to 2021, the distribution of goji berry cultivation bases in Zhongning County and its adjacent surrounding counties, including Shapotou District and Haiyuan County of Zhongwei prefecture-level city, and Tongxin County, Hongsipu District, Litong District and Qingtongxia of Wuzhong prefecture-level city was collected through field investigation. Finally, the distributional information of 55 goji berry cultivation bases in the *Daodi* region was collected (**Figure 2**).

The data on soil were obtained from the World Soil Database (Harmonized World Soil Database v1.2, http://www.fao.org/soils-portal/), and converted to the ASCII format using ArcGIS v10.8 (https://www.esri.com/zh-cn/arcgis/). The dataset contained 18 soil variables. Due to the lack of data on future soil layers, we assumed that future soil layers were consistent with the current ones over these short time frames (Zhang et al., 2020a; Huang et al., 2022). The current (averages for 1960 – 1990) and the future 2050s (averages for 2041 – 2060) and 2070s (averages for 2061 – 2080) climate data were obtained from the World Climate Database (WorldClim v1.4, 2.5-minute resolution, https://www.worldclim.org/) and converted to ASCII format using ArcGIS. The climatic dataset contained 19 climate variables. Future climate data were determined based on the Community Climate System Model v4.0 (CCSM 4.0). The mode had four greenhouse gas emission scenarios (RCP 2.6, 4.5, 6.0, and 8.5) from the 5^th^ Emission Report of the Intergovernmental Panel on Climate Change (IPCC) (Moss et al., 2010). Thirty-six environmental variables (17 soil and 19 climate variables) were initially used to construct the MaxEnt model.

#### 2.1.2 Source of maps

The maps [inspection number: GS Jing (2022) 1061] of the Ningxia Hui Autonomous Region, Gansu Province, Inner Mongolia Autonomous Region, and China were downloaded from the Alibaba Cloud Data Visualization Platform (DataV. GeoAtlas, http://datav.aliyun.com/portal/school/atlas/areaselector/) and converted to shapefiles using Mapshapper (https://mapshaper.org/).

### 2.2 Methods

#### 2.2.1 Screening of goji berry distribution points and environmental variables

The spatial analysis function of ArcGIS was used to estimate the distance between the distribution points to reduce the effect of spatial autocorrelation and improve prediction accuracy. Consequently, duplicate points were deleted, and only one point was maintained when the straight-line distance between two points was less than 10 km.

The environmental variables were examined for cross-correlation to avoid multicollinearity and improve the MaxEnt model’s accuracy (Jarnevich et al., 2015). First, Pearson’s correlation between the current environmental variables was analyzed using the correlation function of ENMTools v1.3 (https://github.com/danlwarren/ENMTools/). Second, the environmental variable data and the distribution data of goji berry were imported into MaxEnt v3.4.4 (https://biodiversityinformatics.amnh.org/opensource/maxent/) with 25% of the distribution points in the test dataset and the remaining 75% in the training dataset. A jackknife analysis was performed to measure variable importance. The logistic format and a replicate run type bootstrap were also selected as MaxEnt model features. The model runs were repeated 10 times. The environmental variables with zero contribution were removed. Variables with an absolute value of their correlation coefficient greater than 0.8 were retained only when they reached the highest contribution in the jackknife analysis (Li et al., 2022; Zhao et al., 2022b).

#### 2.2.2 Optimization and evaluation of the MaxEnt model

The regularization multiplier (RM) and feature classes (FCs) are the key parameters affecting the prediction accuracy of the MaxEnt model (Muscarella et al., 2014). The default settings in MaxEnt produce overfitting results and are typically not optimal. We used the Kuenm package (https://github.com/marlonecobos/kuenm/) in R v3.6.3 (https://www.r-project.org/) to calibrate RM and FCs for the optimal MaxEnt (Cobos et al., 2019). The RM parameter was set to 0.1 – 4.0 at an interval of 0.1. The MaxEnt model included five FCs [linear (L), quadratic (Q), hinge (H), product (P), and threshold (T)] and 31 possible FCs combinations. The Kuenm package was applied to evaluate the 1,240 (40 RMs × 31 FCs) candidate modes. The output file (selected_models.csv) was used to select the optimal candidate modes. The best models were selected under current climate conditions according to the following criteria: (1) significant models with omission rates ≤ 5%, and (2) the lowest delta-corrected Akaike information criterion (AICc) of ≤ 2% (Cobos et al., 2019).

The other MaxEnt parameters, which included “Create responsive curves,” “Do jackknife to measure variable importance,” “Out format logistic,” “Random seed,” “Replicated run type bootstrap,” “Add all samples to the background,” and “Write background predictions” were selected for simulation and prediction (Zhao et al., 2022b). The settings for the remaining parameters were default (Zhang et al., 2018a; Wang et al., 2021; Li et al., 2022). The screening data for environmental variables and distribution points were imported into MaxEnt, with 25% of the distribution points used for model testing and the remaining 75% for model training. The model runs repeated 10 times.

The accuracy of the model prediction results was evaluated using the receiver operation characteristic (ROC) area under the curve (AUC) and true skill statistic (TSS) under current climate conditions. The closer the AUC and TSS values were close to 1, indicating a high prediction accuracy (Hou et al., 2023). The model prediction performance was deemed when the AUC and TSS values were greater than 0.9, and 0.75, respectively (Allouche et al., 2006; Franco et al., 2022).

#### 2.2.3 Classification of suitable habitats

The ASCII files output by MaxEnt were reclassified and visualized using ArcGIS. The maximum training sensitivity plus specificity logistic threshold (MTSPS) output by MaxEnt under current climatic conditions was employed to classify these ASCII files into unsuitable and suitable for the *Daodi* goji berry. This threshold is considered simple and effective in determining modeled species’ presence/absence maps (Huang et al., 2022). Areas with suitability values greater than MTSPS were considered suitable for the species (Dai et al., 2023). The distribution areas were divided into unsuitable habitat (0 – MTSPS), lowly suitable habitat (MTSPS – 0.6), moderately suitable habitat (0.6 – 0.7), and highly suitable habitat (0.7 – 1.0) according to the suitable probability using the ArcGIS reclassification function.

#### 2.2.4 Area calculation of suitable habitats

The number of grids in different suitable habitats was counted using ArcGIS. The area of each grid was calculated as follows:


$$\text{Area of each grid}=\frac{\text{Land area of China}}{\text{Grid number of bio}11\text{ in China}}$$

The area of different suitable habitats was calculated as follows:


Area of suitable habitat=(Number of grids in suitable habitat)×(Area of each grid)

#### 2.2.5 Spatiotemporal and centroid changes in suitable habitats

The ASCII files output by MaxEnt were imported into ArcGIS. Then, SDMtoolbox v2.0 (http://www.sdmtoolbox.org/) was employed to convert these ASCII files to binary files (0 unsuitable habitat, 1 suitable habitat) with the threshold value of 0.6. The spatiotemporal changes in the 2050s and 2070s compared with the current and the centroid changes of the moderately and highly suitable habitats under different climate scenarios were analyzed using SDMtoolbox. The spatiotemporal changes in the area were calculated using the method mentioned in Section 2.2.4.

#### 2.2.6 Overlap of suitable habitats under different climate scenarios

The binary files mentioned in Section 2.2.5 were imported into ArcGIS. Then, the plus function in Spatial Analyst Tools was applied to determine the overlap of moderately and highly suitable habitats under current and future climate scenarios. The suitable overlapping area was calculated using the method mentioned in Section 2.2.4.

## 3 Results

### 3.1 Screening results of distribution points and environmental variables, and accuracy of the MaxEnt model

**Figure 3:**
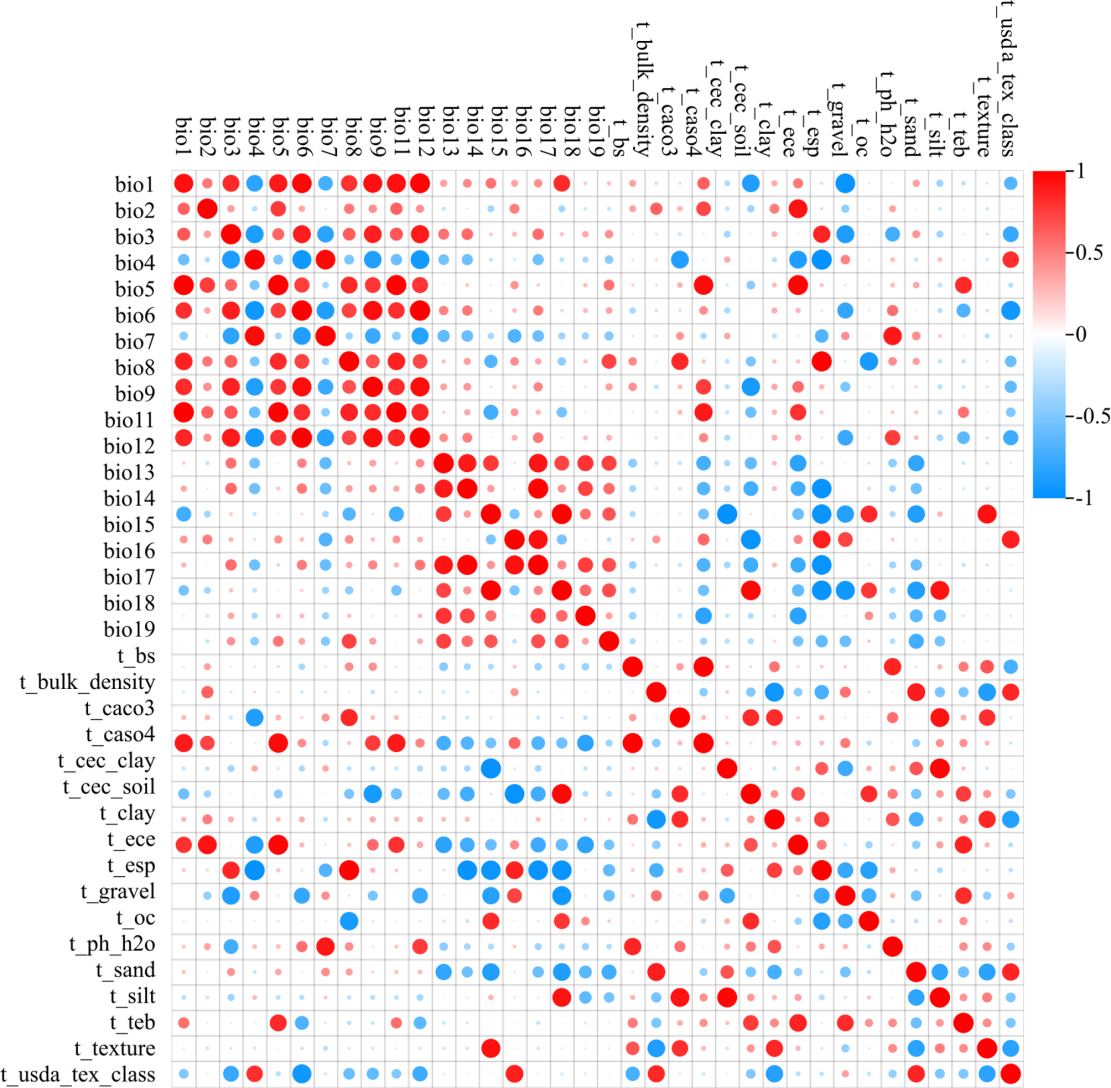
Correlation of environmental variables.

The correlation of the environmental variables was shown in **Figure 3**. After the screening, 26 of 55 distribution points and 14 (five climate variables and nine soil variables) of 36 environmental variables (**Table 1**) were selected for MaxEnt analysis.

**Table 1:** Percent contribution and permutation importance of the screening environmental variables under current climate conditions.

Variable	Description	Percent contribution (%)	Permutation importance (%)
bio11	Mean temperature of coldest quarter	33.4	86.2
bio19	Precipitation of coldest quarter	29.5	1.6
bio18	Precipitation of warmest quarter	10.9	1.9
t_caco3	Topsoil calcium carbonate	10	0
bio14	Precipitation of driest month	8.1	9.3
t_gravel	Topsoil gravel content	4.7	0
bio12	Annual precipitation	1.4	0.9
t_texture	Topsoil texture	0.7	0
t_bs	Topsoil base saturation	0.6	0
t_cec_soil	Topsoil CEC (soil)	0.3	0
t_ece	Topsoil Salinity (Elco)	0.1	0
t_cec_clay	Topsoil CEC (clay)	0.1	0
t_esp	Topsoil Sodicity (ESP)	0.1	0
t_teb	Topsoil TEB	0	0

Based on the screening distribution points and environmental variables, the potential distribution of *Daodi* goji berry was simulated and predicted by the MaxEnt model. When we selected default parameters for the model, the delta AICc was 54.85. Based on the results output by the Kuenm package, when RM and FC were separately set to 2.1 and LQPH, delta AICc was observed to be 0. Therefore, RM = 2.1 and FC = LQPH were the optimal combination for MaxEnt modeling (**Supplementary Table S1**). The 10-repeated average training AUC (**Supplementary Figure S1**) and the average TSS were 0.999 and 0.945, respectively, indicating that the reconstructed model was highly reliable and could effectively predict the *Daodi* goji berry suitable habitats.

### 3.2 Dominant environmental variables influencing the distribution of *Daodi* goji berry

The dominant environmental variables influencing the distribution of *Daodi* goji berry under current climate conditions were determined through their contribution rates and the jackknife of regularized training gain (Sun et al., 2012). The variables with the highest contribution rate to the model were bio11 (33.4%) and bio19 (29.5%), with a cumulative contribution rate of 62.9% and permutation importance of 87.8% (**Table 1**). The results of the jackknife test of variable importance showed that bio11 and bio19, with the highest training gain, were the two critical variables shaping the distribution of the *Daodi* goji berry (**Supplementary Figure S2**).

Based on a comprehensive analysis of the contribution rates and the regularized training gain, the dominant variables influencing the distribution of *Daodi* goji berry were bio11 and bio19. The relationship between the distribution probability and the dominant environmental variables was studied based on the species response curves under current climate conditions. When the potential distribution probability was greater than 0.5473 (MTSPS), the mean temperature (bio11) and precipitation (bio19) of the coldest quarter were from −6.40°C to −2.57°C and less than 13.97 mm, respectively (**Supplementary Figure S3**).

### 3.3 Potentially suitable habitats of *Daodi* goji berry

#### 3.3.1 Potentially suitable habitats under current climate conditions

Under current climate conditions, the actual distribution of goji berry production bases in the *Daodi* region highly matched the MaxEnt prediction results. 92.31% of the screening distribution points were located in suitable habitats, of which 76.92% were in moderately and highly suitable habitats.

**Figure 4:**
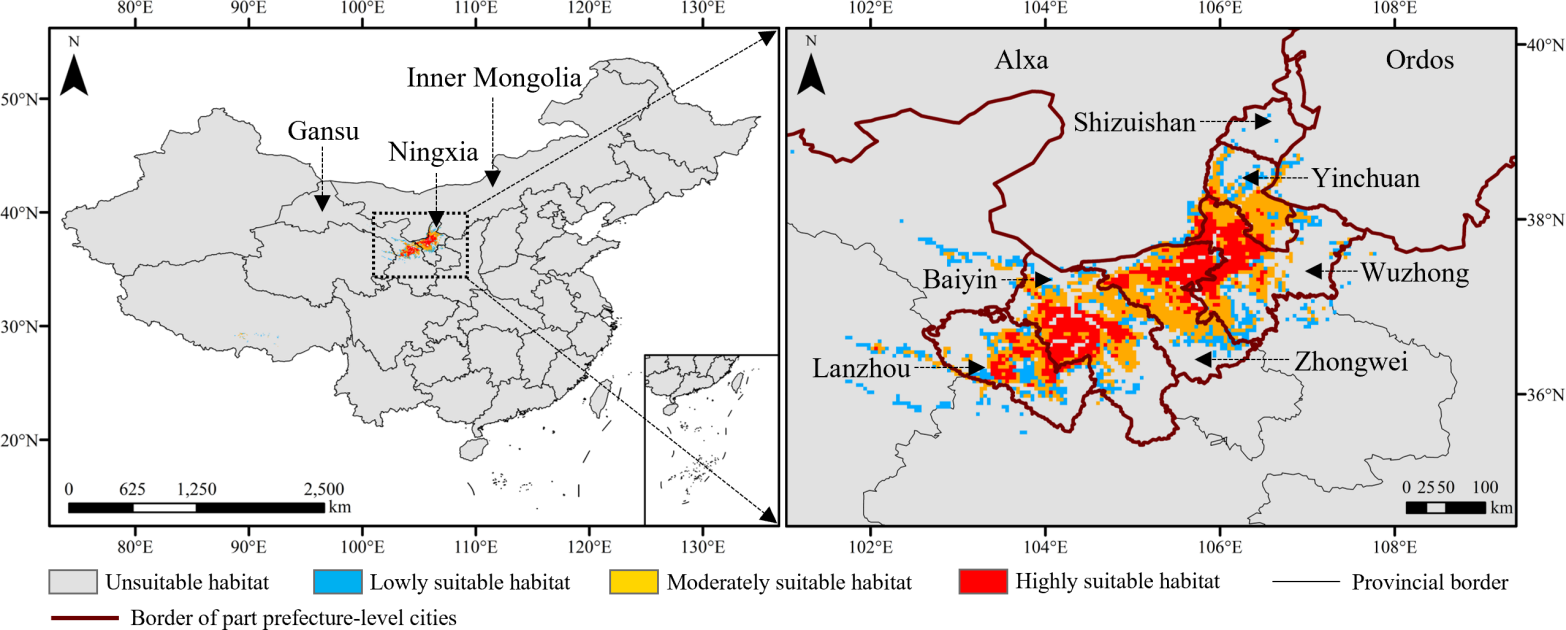
Suitable habitats of the *Daodi* goji berry under current climate conditions.

**Figure 5:**
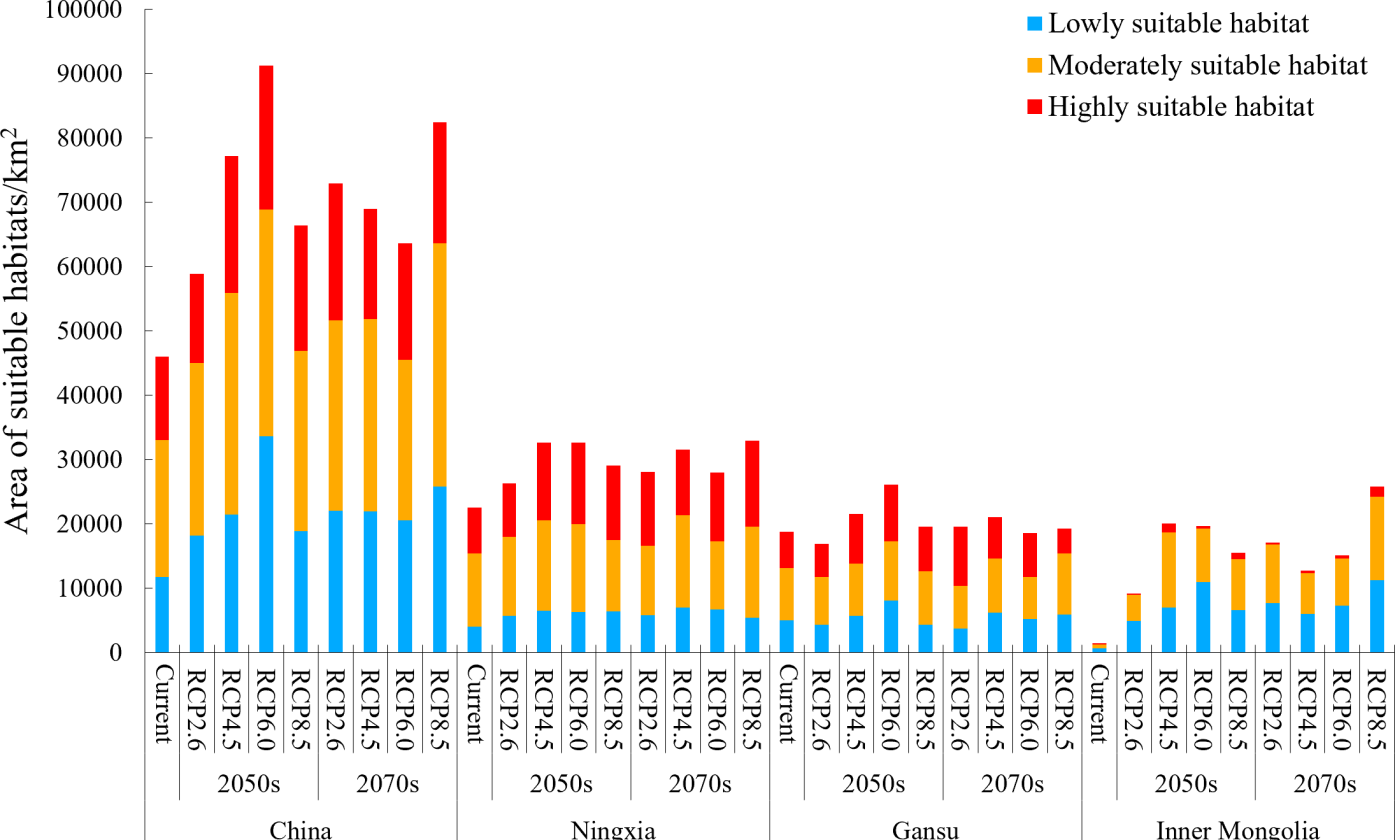
Suitable area of the *Daodi* goji berry under current and future climate scenarios.

The habitats suitable for the growth of *Daodi* goji berry were 45,973.88 km^2^, accounting for 0.48% of China’s land area, which were concentrated in central and western Ningxia province and central Gansu province (**Figures 4**, **5**). The suitable habitats in Ningxia were the largest, accounting for 49.14% of those in China, followed by Gansu (40.86%) and Inner Mongolia (3.27%) (**Figure 5**).

In Ningxia province, the suitable habitats were mainly distributed in Zhongwei (9,160.21 km^2^) and Wuzhong (9,004.66 km^2^) prefecture-level cities, where *Daodi* goji berry was located, accounting for 80.41% of those in Ningxia, followed by Yinchuan (4,338.14 km^2^) (**Figure 4**). In Gansu province, the suitable habitats were mainly located in Baiyin (10,439.18 km^2^) and Lanzhou (6,135.61 km^2^) prefecture-level cities, accounting for 88.22% of those in Gansu. The region with the widest highly suitable habitats was Baiyin (3,785.07 km^2^), followed by Wuzhong (3,508.53 km^2^), Zhongwei (3,214.71 km^2^), Lanzhou (1,762.91 km^2^), and Yinchuan (380.24 km^2^) (**Figure 4**).

#### 3.3.2 Potentially suitable habitats under different future climate scenarios

**Figure 6:**
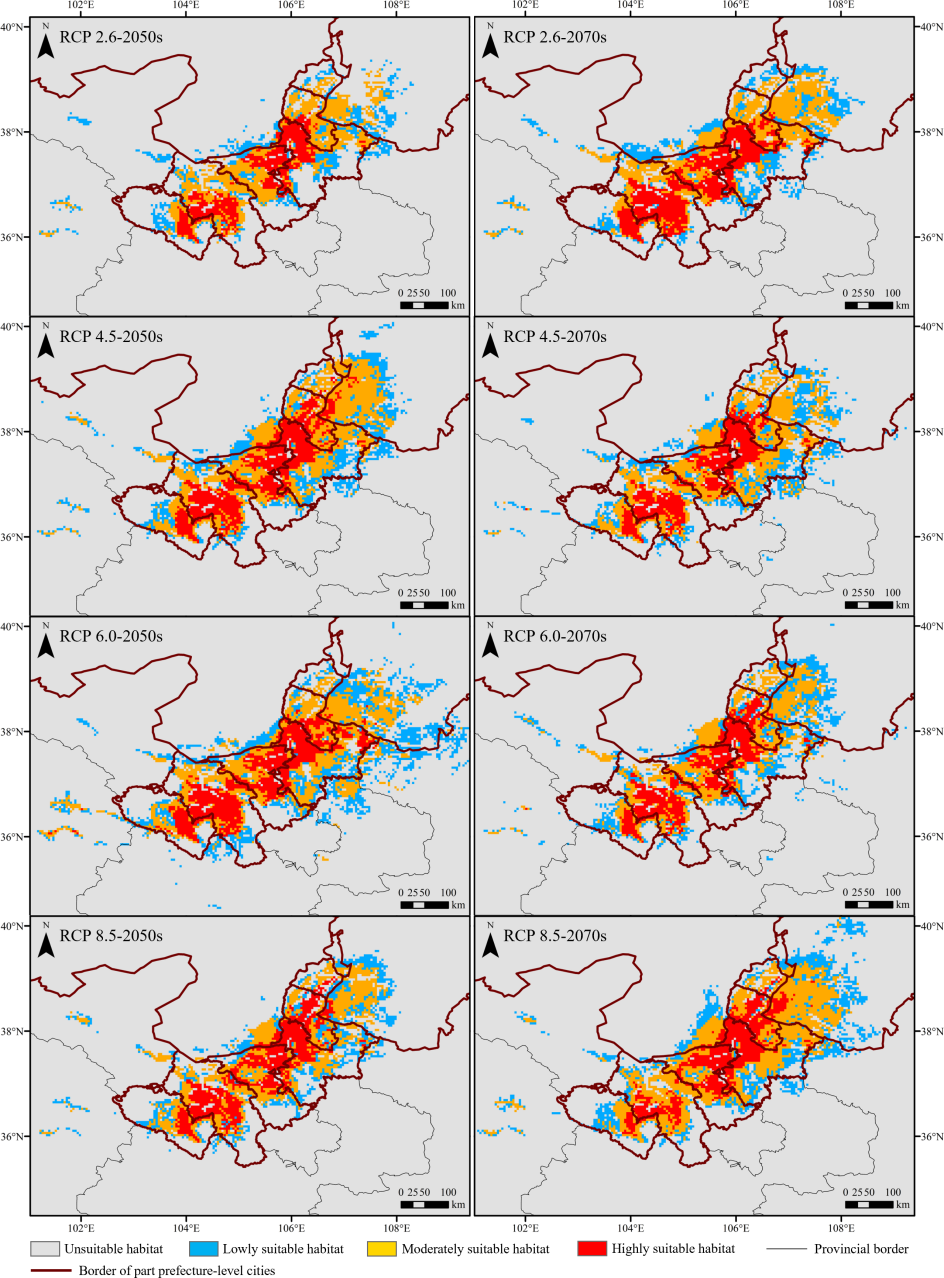
Suitable habitats of the *Daodi* goji berry under different climate scenarios.

Under different future climate scenarios, the area of potentially suitable habitats was higher than that under current climate conditions (**Figure 5**). Additionally, these habitats were mainly distributed in Ningxia, Gansu, and Inner Mongolia provinces (**Figure 6**; **Supplementary Figure S4**). In the 2050s, the area of suitable habitats under RCP 2.6, 4.5, and 6.0 climate scenarios expanded with the increased greenhouse gas emissions, except RCP 8.5. Among the four climate scenarios in the 2050s, the suitable area under RCP 6.0 reached the maximum (91,256.42 km^2^), with an increase of 98.50% compared with the current. However, the changes in suitable area in the 2070s were opposite to those in the 2050s. In the 2070s, the suitable area decreased gradually under RCP 2.6, 4.5, and 6.0 but reached the maximum (82,459.17 km^2^) under RCP 8.5. The highly suitable area under different future climate scenarios was higher than the current ones, mainly distributed in Ningxia and Gansu provinces, and reached the maximum under RCP 6.0 (22,416.59 km^2^) in the 2050s and 2.6 (21,224.03 km^2^) in the 2070s (**Figure 5**).

In Ningxia, the area of total suitable and highly suitable habitats for the distribution of *Daodi* goji berry under future climate scenarios was higher than the current ones, and reached the maximum under RCP 8.5 (suitability: 32,907.62 km^2^; highly suitability: 13,290.94 km^2^) in the 2070s (**Figure 5**). The highly suitable habitats were mainly distributed in central and western Ningxia, including Zhongwei and Wuzhong, followed by Yinchuan (**Figure 6**).

In Gansu, the suitable habitats’ location was consistent with the current and mainly distributed in Baiyin and Lanzhou (**Figure 6**). The total suitable and highly suitable area reached the maximum under RCP 6.0 (suitability: 2,607.95 km^2^; highly suitability: 8,814.54 km^2^) in the 2050s, and reached the maximum under RCP 4.5 (suitability: 2,1051.20 km^2^) and 2.6 (highly suitability: 9,263.91 km^2^) in the 2070s, respectively (**Figure 5**).

In Inner Mongolia, the suitable habitats under future climate scenarios expanded dramatically and increased to 6.14 – 17.18 times compared with the current (**Figure 5**), which mainly concentrated in the southwest Ordos prefecture-level city bordering with Ningxia, followed by Alxa (**Figure 6**). The area of highly suitable habitats reached a maximum of 1,590.07 km^2^ under RCP 8.5 in the 2070, which was 8.36 times larger than the current.

#### 3.3.3 Spatiotemporal and centroid changes in moderately and highly suitable habitats

**Figure 7:**
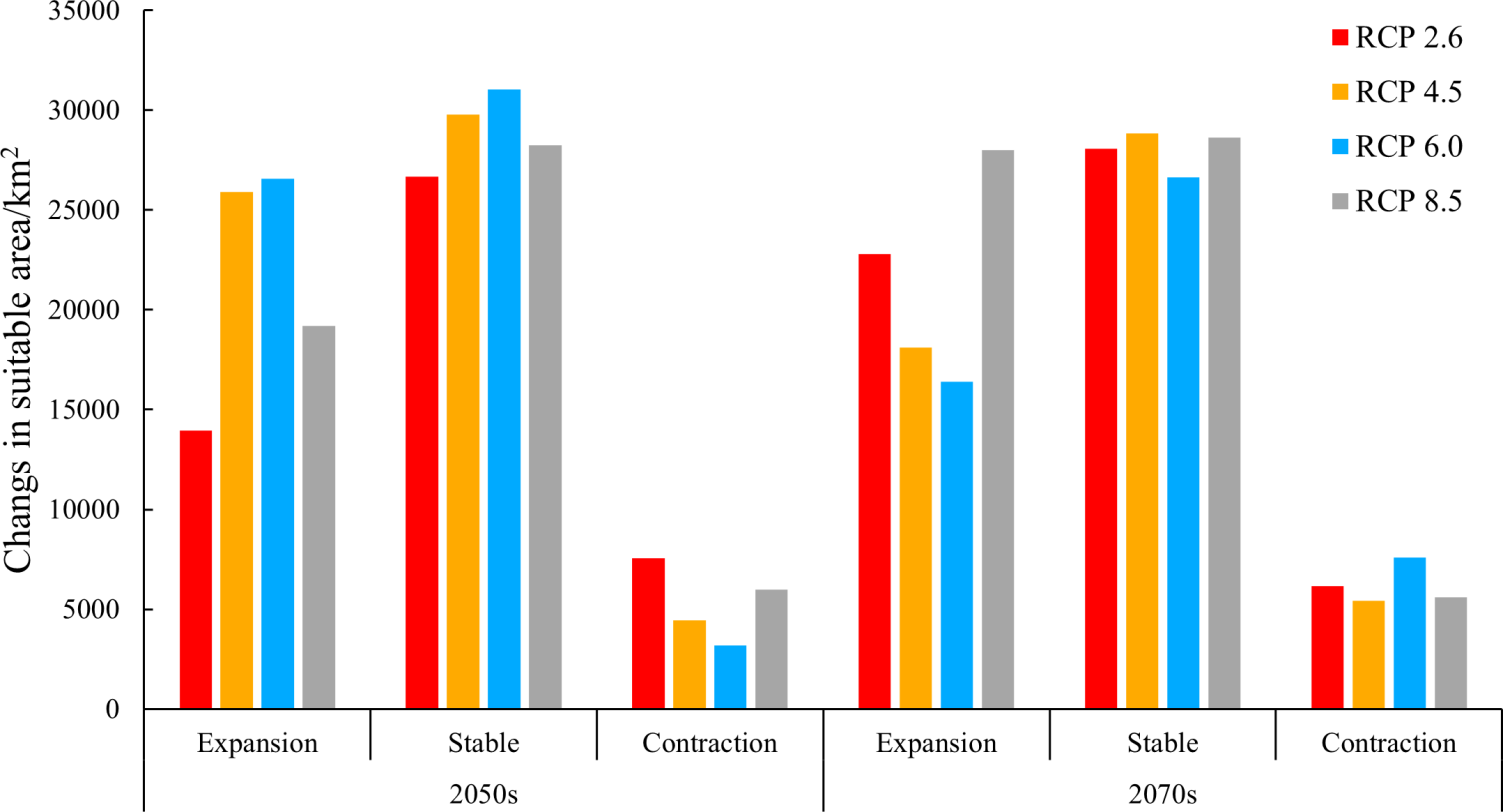
Changes in the area of moderately and highly suitable habitats under different future climate scenarios compared with those under the current climate conditions.

**Figure 8:**
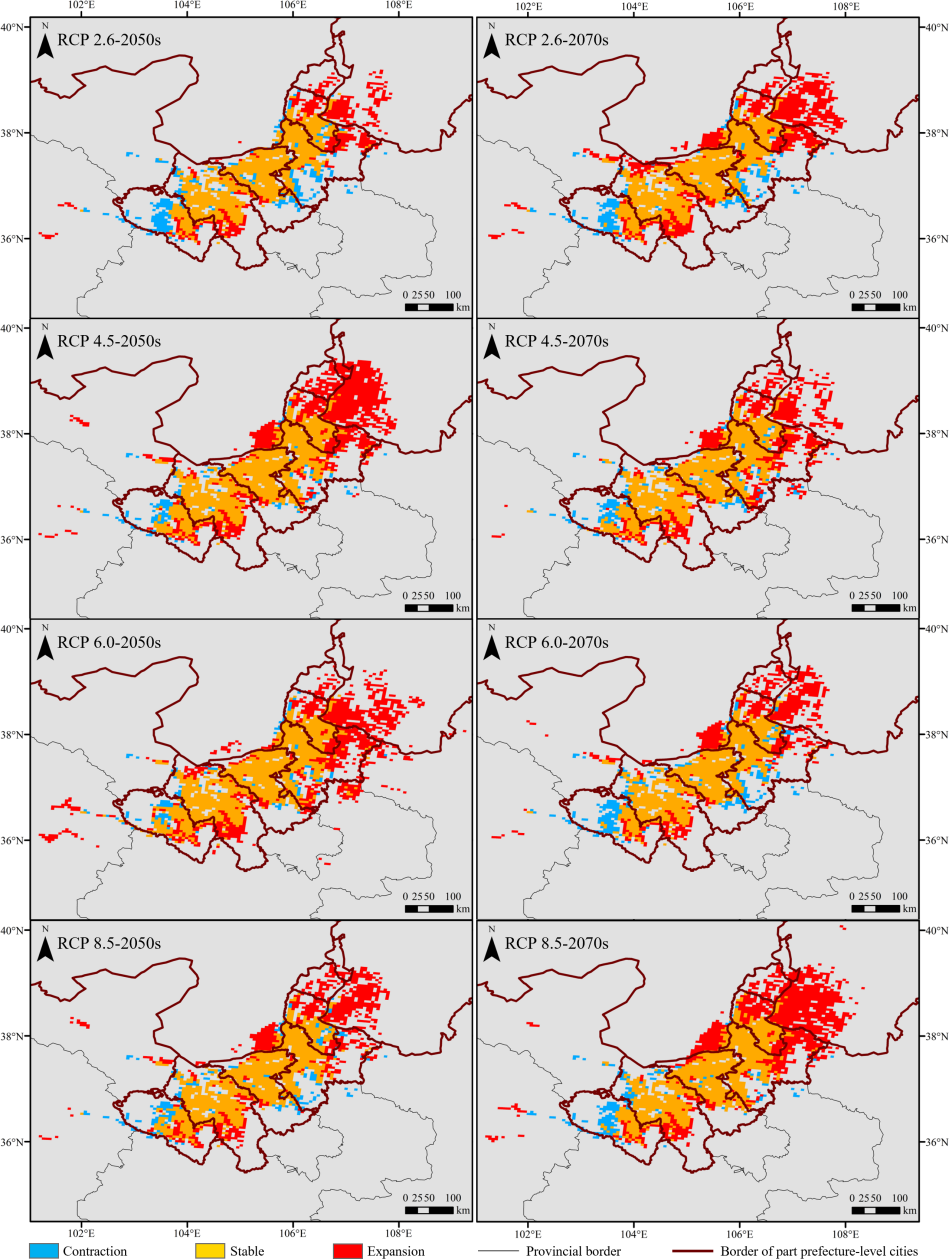
Spatiotemporal changes in moderately and highly suitable habitats under different future climate scenarios compared with those under current climate conditions.

Compared with the current climate conditions, the expansion area of moderately and highly suitable habitats under future 2050s and 2070s climate scenarios was all higher than the contraction, with an increase of 0.84 – 7.31 fold (**Figure 7**). The expansion regions were mainly distributed in the northeast of the current moderately and highly suitable ranges, including Ordos, Wuzhong, Yinchuan, and Shizuishan prefecture-level cities (**Figure 8**). On the contrary, the contraction regions were mainly located in Lanzhou prefecture-level city, southwest of the current moderately and highly suitable ranges. Under different future climate scenarios, the expansion area reached the maximum (28,016.41 km^2^) under RCP 8.5 in the 2070s and was close to the unchanged (stable) area (28,638.62 km^2^) of moderately and highly suitable habitats (**Figure 7**).

**Figure 9:**
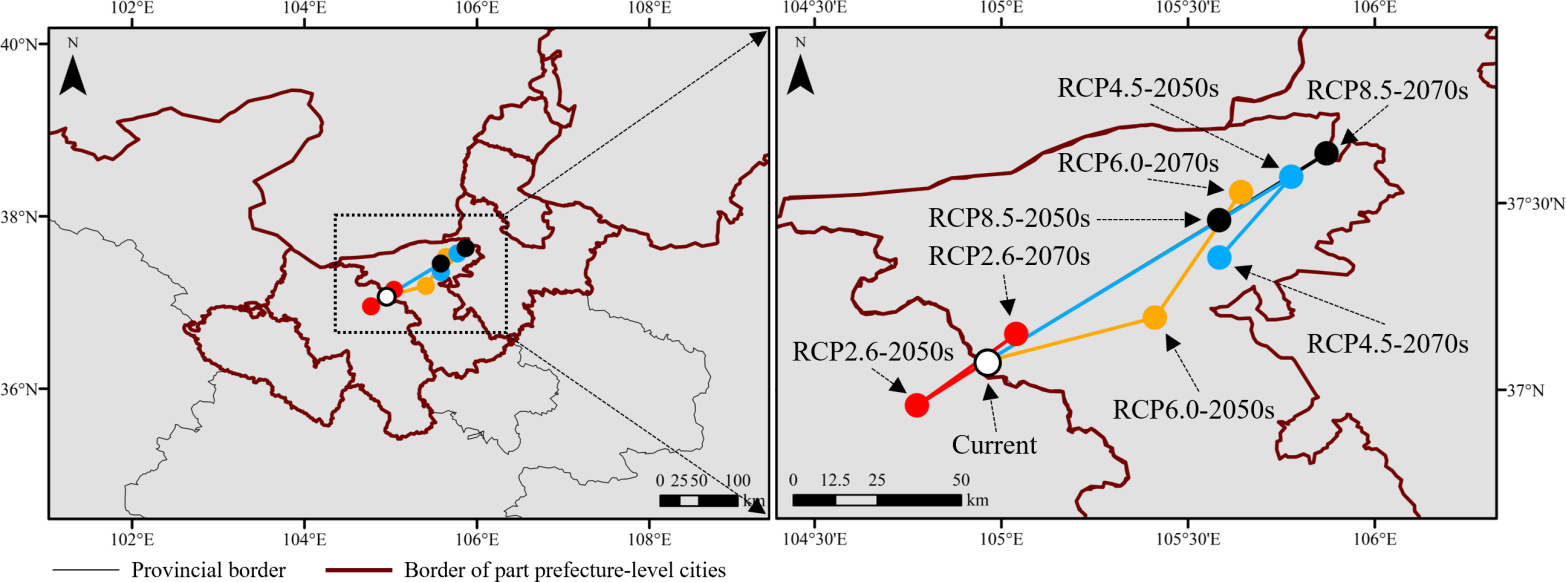
Centroids of moderately and highly suitable habitats under current and future climate scenarios.

Under current climate conditions, the moderately and highly suitable habitat centroid was located in Zhongwei (104.96°E, 37.08°N), Ningxia province, bordering Baiyin, Gansu province (**Figure 9**). Under future climate scenarios, the distributional centroids shifted northeast but still located in Zhongwei, except for RCP 2.6 (104.77°E, 36.65°N) in the 2050s, which was projected to shift southwest into Baiyin. The distance between the centroid of RCP 8.5 (105.87°E, 37.63°N) in the 2070s and the current was 101.16 km, which was larger than the others (**Figure 9**).

#### 3.3.4 Overlap of suitable habitats under different climate scenarios

**Figure 10:**
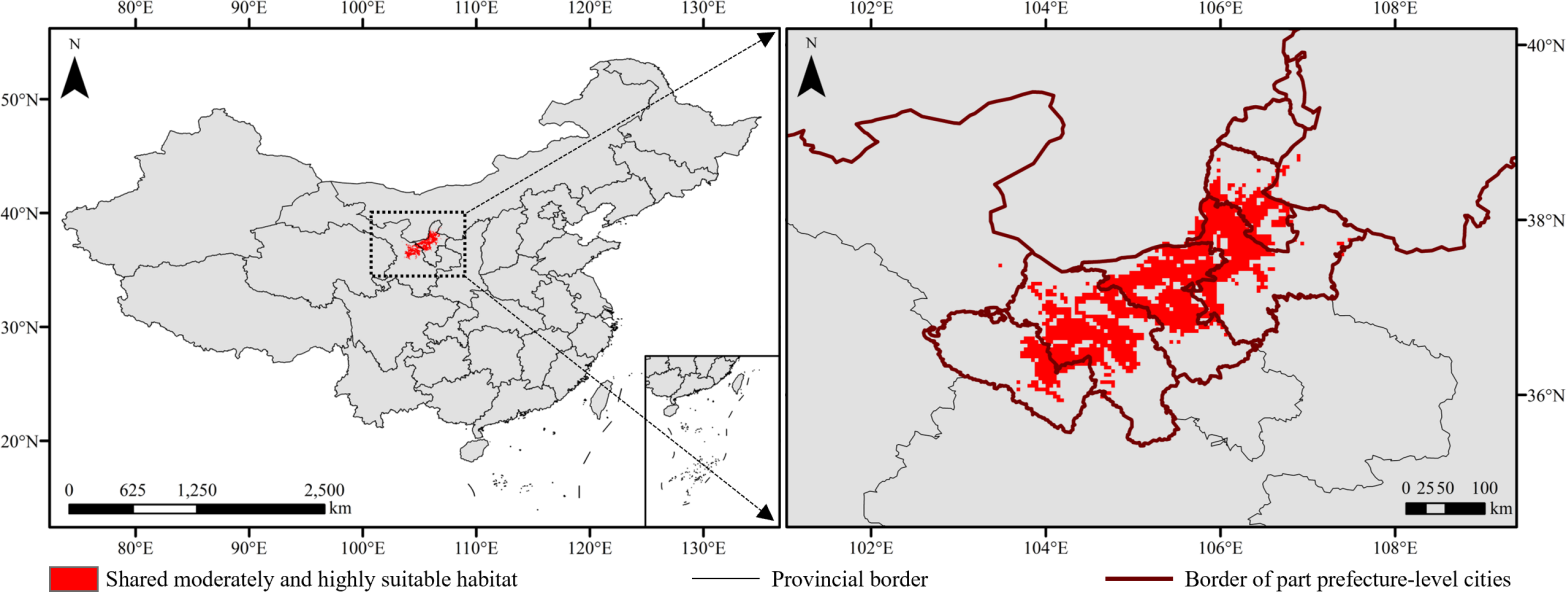
Moderately and highly suitable overlapping habitats under climate change.

**Figure 11:**
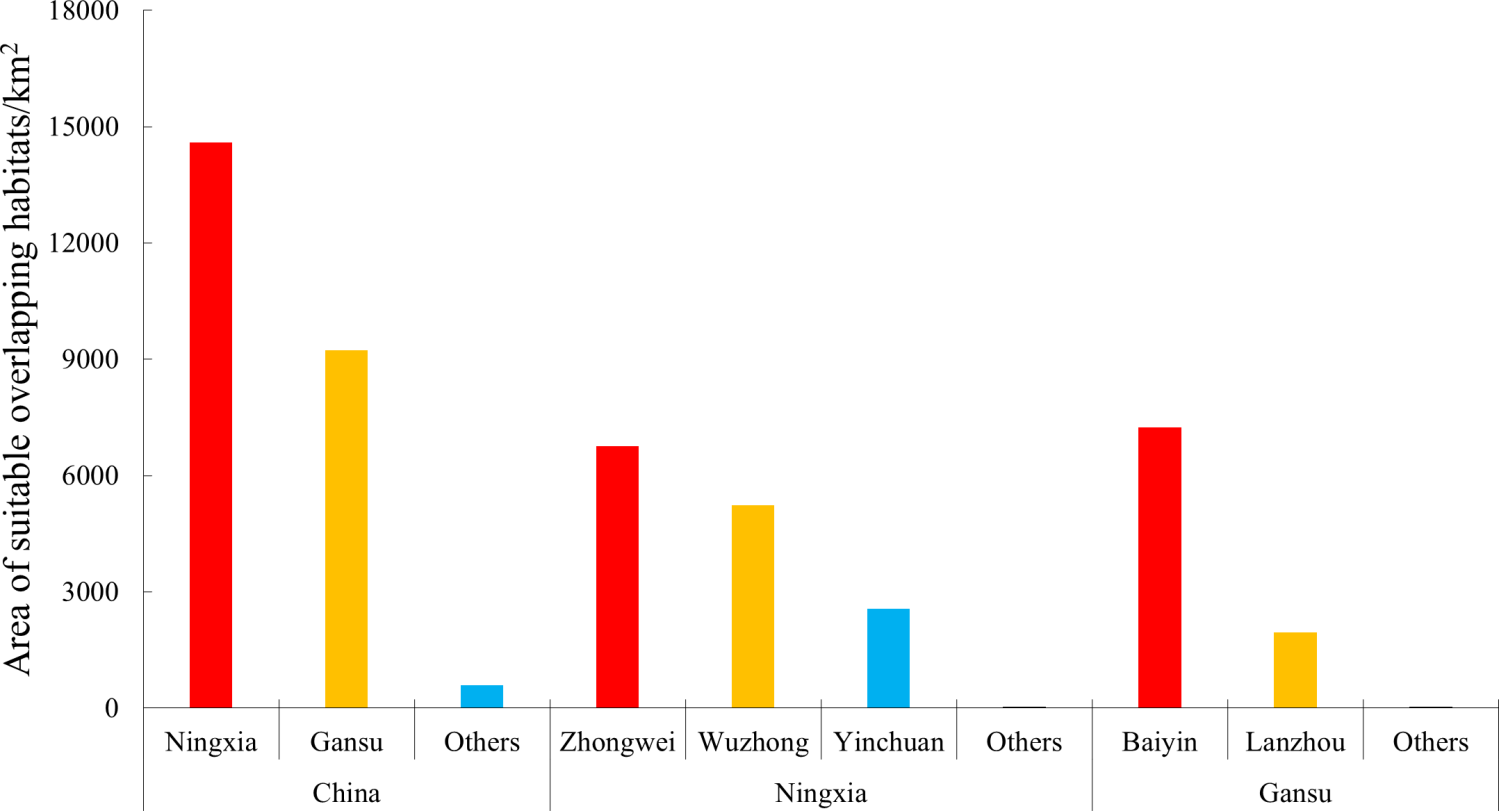
Area of moderately and highly suitable overlapping habitats under climate change.

Under current and future climate scenarios, the moderately and highly suitable overlapping habitats were mainly concentrated in Ningxia (14,587.20 km^2^) and Gansu (9,229.34 km^2^) provinces, accounting for 59.77% and 37.82% of those of China, respectively (**Figures 10**, **11**). In Ningxia, the overlapped regions were mainly distributed in Zhongwei (6,757.81 km^2^) and Wuzhong (5,236.87 km^2^) prefecture-level cities, followed by Yinchuan (2,557.94 km^2^). In Gansu, the overlapped regions were mainly distributed in Baiyin (7,241.75 km^2^) prefecture-level city, followed by Lanzhou (1,953.03 km^2^) (**Figure 11**).

## 4 Discussion

In this study, MaxEnt and ArcGIS were used to predict the suitable habitats of the *Daodi* goji berry in China and analyze its changes under different greenhouse gas emission modes based on the environmental characteristics of the *Daodi* region. Our study was significant in guiding the scientific cultivation and meeting the supply of high-quality goji berry. As concentrated distribution points and cross-correlations among the environmental variables led to the overfitting of the MaxEnt model (Zhang et al., 2018a; Wang et al., 2023), we used the spatial analysis function of ArcGIS and ENMTools to screen the distribution points and environmental variables to avoid these issues. Moreover, to improve the MaxEnt prediction accuracy, the key parameters RM and FC were optimized using the Kuenm package. The AUC and TSS values of the optimized model were all larger than 0.90, and more than 90% distribution points were located in the suitable habitats, indicating that the simulation and prediction of the model were excellent and reliable. Thus, the model could be used to predict the geographical distribution of the *Daodi* goji berry.

The prediction results showed that the potentially suitable habitats for the *Daodi* goji berry were mainly distributed in central and western Ningxia province and central Gansu province bordering western Ningxia under current climate conditions, which were consistent with the known distribution of the cultivated goji berries (Xu et al., 2014). These regions were located in the upper reaches of the Yellow River with similar environmental characteristics and suitable for cultivating goji berries (Li et al., 2019a). Climate warming affects the adaptability of plants, causing the expansion of thermophilic plants and the retreat of psychrophilic plants to the Antarctic and Arctic (Parmesan and Yohe, 2003; Hegland et al., 2009; Liang et al., 2018). Our results showed that the area suitable for the *Daodi* goji berry under future climate scenarios was all higher than the current, and the distributional centroids mainly shifted to the northeast of China. Given that goji berries are infinite inflorescence plants, the warming climate will extend the growth period, resulting in increased yields and benefiting the accumulation of active ingredients such as polysaccharides (Li et al., 2019b; Xu et al., 2023). Thus, we believe climate warming is conductive to *Daodi* goji berry cultivation.

The environmental conditions are the dominant factors influencing the physiology, distribution, and phenology of plants (Xue et al., 2021; Wang et al., 2023). Therefore, climate change may result in the migration of CMMs’ *Daodi* region (Peng et al., 2013). We identified that the moderately and highly suitable overlapping habitats of *Daodi* goji berries were primarily distributed in Baiyin, Zhongwei, and Wuzhong. Under climate change, these areas consistently maintained their suitability for cultivating *Daodi* goji berries. Zhongning County, the focal region for cultivating *Daodi* goji berries, is situated at the junction of Zhongwei and Wuzhong (Huang et al., 2020). Based on our findings, we propose that climate warming will not lead to the migration of *Daodi* goji berry location.

Among all the environmental variables, climate, including temperature and precipitation, is the most crucial factors controlling plant regeneration and distribution (Thuiller, 2007). Our results showed that the contribution rate (83.3%) and the permutation importance (99.9%) of climate variables were much higher than the soil, indicating that climate had a much greater impact on *Daodi* goji berry distribution than soil. This characteristic was also found in many other plants (Hageer et al., 2017). For example, the dominant environmental variables influencing the distribution of mountain pepper *Litsea cubeba* (Lour.) Pers. were precipitation of the driest quarter, annual precipitation, temperature annual range, and min temperature of the coldest month instead of soil variables (Shi et al., 2023). In temperate ecosystems, winter ecological processes are important drivers of vegetation and ecosystem functioning (Kreyling, 2010). In this study, the mean temperature of the coldest quarter (bio11) was the most crucial environmental variable influencing the distribution of the *Daodi* goji berry, and the suitable range was −6.40°C – −2.57°C; this was closely related to the habits of the shrub, which was mainly distributed in the arid and cold regions of northwest China (Lu et al., 2020). It has been proved that goji berry was mainly distributed in the suitable range of −10°C to 10°C for the mean temperature in winter (Wang et al., 2022b). When the temperature was below -15°C, the germination of its dormant branches would decrease significantly (Qi et al., 2016).

We found that Baiyin, Gansu province, was always moderately and highly suitable for *Daodi* goji berries under climate change. Previous studies have shown that although the longitudinal and transverse diameters of goji berries from Baiyin were larger than those from Zhongning County, there were no significant differences in the concentrations of polysaccharides, betaine, carotenoids, flavonoids, total phenols, trace elements, etc. (Lu et al., 2019; Wang et al., 2019a; Wang, 2020). Moreover, Li et al. (2019a) showed that the quality (polysaccharides, protein, vitamins, and naringin) regionalization of goji berry produced in Baiyin was highly consistent with that in Zhongning. Therefore, we suggest that goji berry produced in Baiyin could be used to relieve the insufficient supply of *Daodi* CMMs. According to the concept of *Daodi* CMMs in the Law of the People’s Republic of China on TCM, the production areas of *Daodi* CMMs (*Daodi* regions) were selected through long-term clinical practice and remained relatively stable. Therefore, we suggest that this region should be included when demarcating the boundary of the *Daodi* region of goji berry in the future.

In recent years, the cultivation area of goji berry in Qinghai Province has been constantly expanding, primarily concentrated in the Qaidam Basin of China’s Qinghai-Tibet Plateau. As a result, Qinghai has emerged as the largest cultivation province in China, capturing a market share of 38.94% (General Office of the People's Government of Qinghai Province, 2022), and plays a crucial role in the development of goji berry industry. For the regional unique environmental characteristics, the size and yield of Qaidam Basin-produced goji berries are 1.5 – 2.0 times larger than those from the *Daodi* region, with lower harvesting costs (Wang, 2020; Wang et al., 2020). Our prediction results demonstrated that the Qaidam Basin was unsuitable for *Daodi* goji berry distribution. This did not mean that the efficacy of Qaidam Basin-produced goji berries was lower than those from the *Daodi* region. Wang et al. (2020) found that the contents of metabolites, such as organic acids, alkaloids, and polyphenols, in Qaidam Basin-produced goji berry were lower than *Daodi* region, while the contents of lipids and amino acids were higher. However, other research results demonstrated that there was no significant difference in the contents of carotenoids, total phenols, rutin, and total polysaccharides between the two regions (Lu et al., 2019; Yossa Nzeuwa et al., 2019). Variations in sample collection sites, harvest periods, and varieties might cause such differences. Nonetheless, it is widely acknowledged that Qaidam Basin-produced goji berries are more suitable for fruit consumption compared to those from the *Daodi* region, owing to their larger size, higher monosaccharides, and lower pesticide residue (Wang et al., 2019a; Wang et al., 2020). Due to the complexity and diversity of the chemical components of CMMs, different components have synergistic, antagonistic, or neutral effects on each other. Therefore, it is not scientifically reasonable to assess the quality of CCMs according to the content of some active components (Liu et al., 2016; Zhang et al., 2018b; Zhang et al., 2020b). Evaluating the quality of goji berries from different regions based on clinical efficacy is significant for guiding scientific cultivation and differential development.

In this study, the current and future potential habitats suitable for goji berry were screened based on the environmental characteristics of the *Daodi* region for the first time, which was critical in addressing supply shortage and guiding the cultivation of *Daodi* goji berry. However, different cultivation managements are used in other regions, such as the kinds and doses of fertilizers, which may also cause changes in the synthesis of metabolites (Chung et al., 2010; Shi et al., 2019). Therefore, it is necessary to widely collect goji berries from the *Daodi* region and its quasi-regions, and conduct a comprehensive and comparative evaluation of their quality based on massive active components and clinical efficacy.

## 5 Conclusion

Searching for potential regions suitable for cultivating *Daodi* goji berry is significant in meeting the market demands for high-quality medicinal materials. These regions were predicted based on the selected environmental characteristics of the *Daodi* region using the optimized MaxEnt model. Our results indicated that the prediction of the optimized model was highly accurate, and the most important variable affecting the distribution of *Daodi* goji berries was the mean temperature of the coldest quarter. The suitable habitats were mainly distributed in central and western Ningxia and central Gansu at present and would be expanded to the northest of the current ranges under future climate scenarios. The moderately and highly suitable overlapping habitats were mainly located in Baiyin, Zhongwei, and Wuzhong under climate change. Baiyin, adjacent to the *Daodi* region, was always suitable for *Daodi* goji berry distribution at present and in the future. We suggest that climate warming is beneficial to the cultivation of *Daodi* goji berries but has little influence on the *Daodi* region’s location, and the goji berries produced in Baiyin could be used as *Daodi* medicinal materials to meet market demands.

## Data availability statement

The original contributions presented in the study are included in the article/**Supplementary Material**. Further inquiries can be directed to the corresponding author.

## Author contributions

JL: Conceptualization, Formal analysis, Funding acquisition, Investigation, Methodology, Software, Writing – original draft. CD: Conceptualization, Formal analysis, Methodology, Supervision, Writing – review & editing. GD: Investigation, Resources, Writing – review & editing. ZW: Investigation, Resources, Writing – review & editing. YZ: Investigation, Resources, Writing – review & editing. GF: Funding acquisition, Software, Supervision, Writing – review & editing, Project administration.
