# Supplementary material for: Potentially suitable habitats of Daodi goji berry in China under climate change
Source: Front Plant Sci. 2024 Jan 9;14:1279019. doi: 10.3389/fpls.2023.1279019 (PMC10803630; doi:10.3389/fpls.2023.1279019)
Supplement: Supplementary file 1 [file DataSheet_1.docx]

SupplementaryMaterials

**Table S1** Selected models output by the Kuenm package

| Model | Mean_AUC_ratio | pval_pROC | Omission_rate_at_5% | AICc | delta_AICc | W_AICc | num_parameters |
| --- | --- | --- | --- | --- | --- | --- | --- |
| M_2.1_F_lqph_Set_1 | 1.99 | 0 | 0 | 428.42 | 0 | 0.13 | 11 |
| M_1.6_F_lqph_Set_1 | 1.99 | 0 | 0 | 428.68 | 0.26 | 0.12 | 12 |
| M_3.2_F_lqph_Set_1 | 1.99 | 0 | 0 | 428.73 | 0.31 | 0.11 | 9 |
| M_2.7_F_lqph_Set_1 | 1.99 | 0 | 0 | 429.35 | 0.93 | 0.08 | 10 |
| M_3.3_F_lqpth_Set_1 | 1.99 | 0 | 0 | 429.72 | 1.30 | 0.07 | 9 |
| M_2.2_F_lqph_Set_1 | 1.99 | 0 | 0 | 429.73 | 1.32 | 0.07 | 11 |
| M_2.8_F_lqpth_Set_1 | 1.99 | 0 | 0 | 430.23 | 1.81 | 0.05 | 10 |


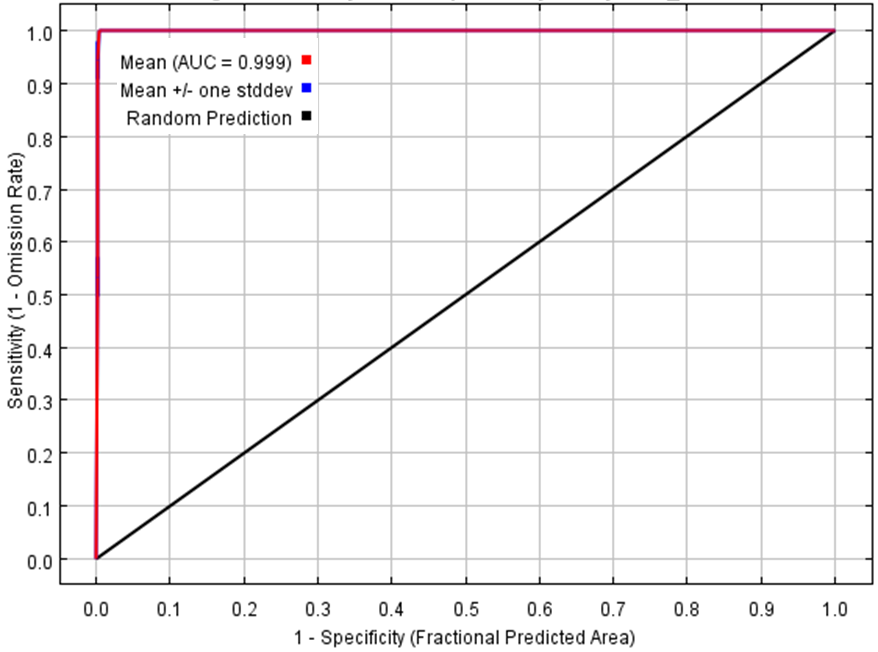


**Fig. S1** Receiver operating characteristic (ROC) curve of the *Daodi* goji berry


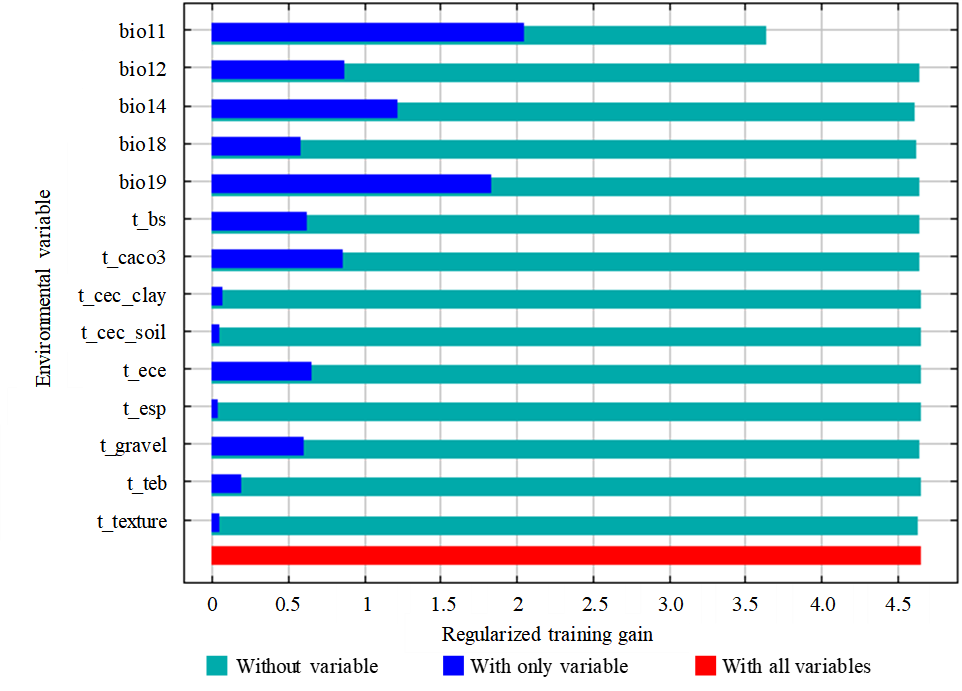


**Figure S2** Jackknife test of variable importance for the MaxEnt model of the *Daodi* goji berry distribution


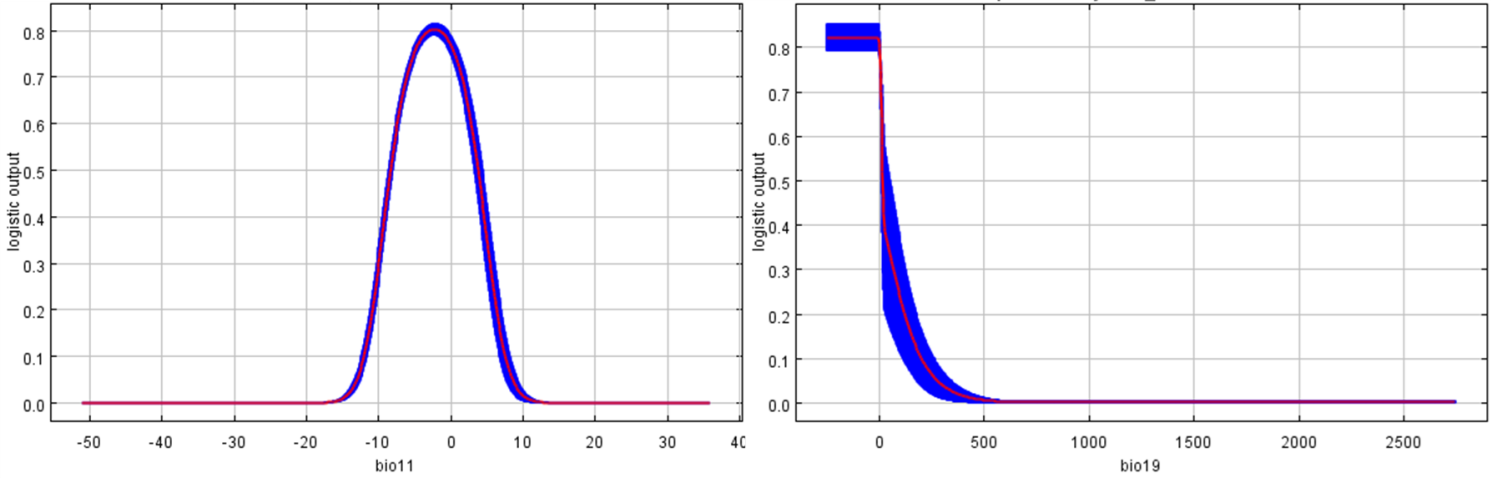


**Fig. S3** Response curves of the *Daodi* goji berry distribution probability to the dominant environmental variables.


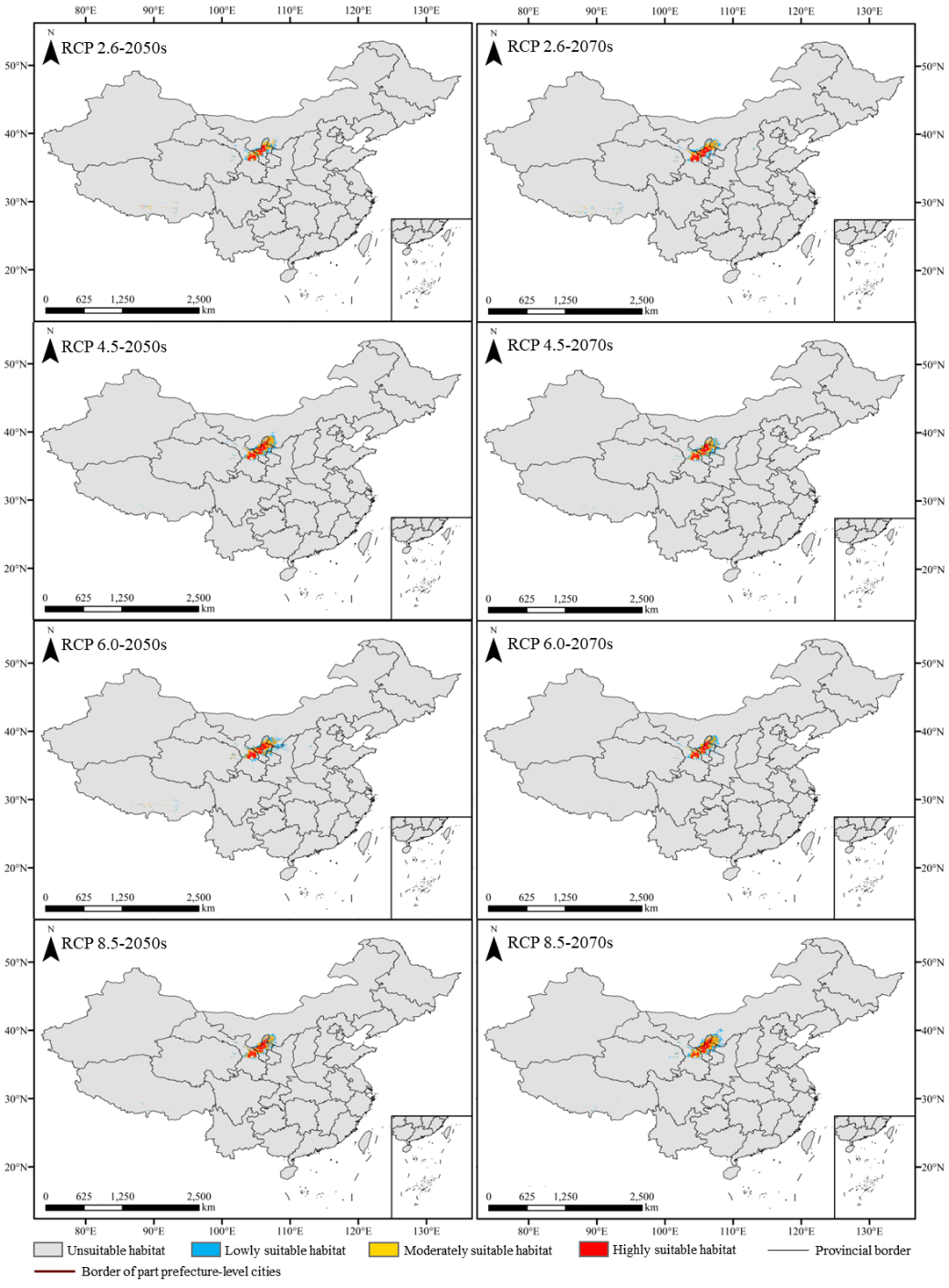


**Fig. S4** Suitable habitats of the *Daodi* goji berry under different future climate scenarios.
